# On Moral and Criminal Epidemics

**Published:** 1856-04-01

**Authors:** 


### Art. VI.—ON MORAL AND CRIMINAL EPIDEMICS.

Whilst the science of teratology was still young and unrecognised, Geoffroy St. Hilaire was one day told by a friend of a wonderful fœtal monstrosity which had just been shown him. "Did you sèe at the same time," asked Geoffroy, "the abortive placenta and umbilical cord of the second fœtus?" "Then you have seen it?" asked his friend. "No; but these are the *necessary* and *inevitable* conditions of an *abnormal* development such as you describe."

The philosopher recognises no accident. To him, there is no phenomenon without *a cause*, an antecedent adequate to its production; no cause but such as is reducible to *law*. He sees alike in the *normal* progress, and in the apparently *exceptional* conditions of the physical and moral world, only illustrations of law and order. The law may appear to be broken, nay, controverted by irregularities; the order may seem to be disturbed by disorders; anomalies may present themselves;—yet in all this he sees but evidence of wider grasp and adaptability; of general principles illustrated under conditions not yet investigated, yet susceptible of being so: the anomaly he knows to be only such in reference to his own finite powers and intelligence; he even retains his conviction, a conviction which affords the only stable foundation for all science, that similar elements, reacting under similar conditions, will produce similar results; and his confidence, that the same power which regulates the succession of day and

Sir Henry Holland has published some admirable papers—On the Effects of Mental Attention on Bodily Organs, in his "Medical Notes and Reflections," and in his chapters on Mental Philosophy, well worthy of the attention of the medical philosopher and observant practitioner.

night, of seed time and harvest, is in operation to "guide the whirlwind and direct the storm."

Does an earthquake spread ruin and devastation over a district—does famine or pestilence exhale its baleful influence over a continent—does a comet glare threateningly upon the earth for a time, and pass away into illimitable space—does the sea swallow up the dry land, or the land encroach upon the sea,—in all this he sees, not the evidence of any new and unknown, but the manifestations of the universal law, acting under conditions as yet imperfectly known to him.

Lastly, does war decimate whole kingdoms, or a moral blight pass over and corrupt a community or a nation; he knows that the passions, impulses, appetites, instincts, prejudices, and weaknesses of man are, as they ever were, the source of all moral disturbances. The elements are constant, though their combinations may be variable. Hence the *history* of yesterday is the *interpretation* of to-day, the *prophecy* of to-morrow. With this conviction of the constancy of the relation between cause and effect ineradicably fixed in the mind, he boldly, yet cautiously, sets about the investigation of these apparently *irregular* phenomena, and the conditions under which they occur. He collects and compares numbers of similar and analogous facts, he considers carefully the powers which are proximately operative in their production, he separates the casual from the universal, the essential from the adventitious, and analyses the whole on strictly inductive principles.

And great is his reward! Not only are the irregularities themselves reduced to system and order, but, in their turn, they are made to contribute their quota to the knowledge and definition of the very laws themselves from which they seemed to err. It was by observation on such principles as these of the abnormal developments of animal structure, that Geoffroy not only constructed the science of teratology, but also laid the foundation for the discovery and definition of the true archetype of the osseous skeleton; it was by analysis of the *irregularities* of the pendulum that the figure of the earth was determined; it was by the observance of what were at first deemed to be *casualties*, that polarized light was discovered, and all the laws of optics defined and advanced. But perhaps the most striking illustration of this principle that the world has ever witnessed has been presented during the last few years, in the discovery of the planet Neptune. Certain *irregularities* in the motions of Saturn and Herschel had long been observed, which were of so peculiar a nature that it even began to be conjectured that at the confines of our system *law* was not so certain in its operations as near the centre. It was evident, however, that this view, if received, would tend to sap the foundation of all science; and men like Leverrier and Adams, who were content to recognise no effect without a definite and sufficient cause which would inevitably and invariably produce the phenomenon, boldly hypothecated the existence of such a cause; and by pursuing a chain of inductive and mathematical reasoning and analysis, which appears almost superhuman, they were enabled ultimately to point their telescope to that part of the heavens where the disturbing body ought to exist—*where it did actually exist*, and so to extend the knowledge of our planetary system twice as far into space as before.

The aspect of the present times leads us anxiously and earnestly to inquire, whether some similar system of investigation may not be applied with advantage to the solution of the startling problems which are everywhere presented to us. The science of Sociology is new and imperfect; yet we are sure that it will afford no exception to the general rule which obtains in all; that, if perfected, it must be through a careful observation of its abnormal, or exceptional, as well as its normal phenomena. Nothing is stronger than the contrast between mind and matter, as to their essential nature; but, on the other hand, nothing is more striking than the correspondence in their mode of development, and in the laws which they mutually obey—such correspondence perhaps arising in some measure from the fact, that mind is only manifested through its connexion with matter, and also in many cases from the overpowering influence which each in turn exerts upon the other. As the body has its condition of health, including many gradations of energy and power, so the mind has *its* normal state, extending from the verge of imbecility to the intelligence almost godlike; as the body is affected by diseases of excitement or depression, so the mind has its passions, its mania, its melancholy; as plague and pestilence attack and hurry off their thousands and tens of thousands at one time, so to an equal extent does a more terrific blight than this pass over a country or a continent, at variable and uncertain periods in the history of man, changing the whole aspect of his moral nature, and converting what was once the image and likeness of God into the semblance of a fiend. At one time the spirit of (falsely so called) *religious* controversy will arouse the most ferocious passions of which human nature is susceptible, provoking mutual persecutions, bloodshed, and wars; at another, an epidemic of resistance to constituted authority will spread over half a world (as in the year 1848) rapid and simultaneous as the most virulent bodily disorder. Again is the *collective character* of mental phenomena illustrated by an anomalous psychological condition invading and dominating over thousands upon thousands, depriving them of everything but automatic action, and giving rise to the popular opinion of demoniacal possession—an opinion in some sense justified by the satanic passions, emotions, and acts which accompany the state. At one period, the aggregate tendency is to retirement and contemplation; hence the countless votaries of monachism and anachoretism; at another the mania is directed towards *action*, having for its proposed end some Utopian scheme, equally impracticable and useless; hence the myriads who have forsaken their kindred, their homes, and their country, to seek a land whose stones were gold, or to wage exterminating war for the possession of worthless cities and trackless deserts.

Less disastrous than these, in their influence numerically upon the mass of mankind, perhaps much more so in their demoralizing results, are those cases in which, in the absence of proper moral culture, the seeds of vice and crime appear to be sown under the surface of society, and to spring up and bring forth fruit with appalling rapidity and paralysing succession. Here it is a forgery, bringing ruin upon thousands; there a suicide, the consequence and self-imposed penalty for other crimes. Now a brother's hand is raised against his brother, a son's against his father; now it is the mother who forgets even her natural instincts, and lifts a murderous hand upon her child; and again, the nearest and dearest relation of life—that of husband and wife—is violently severed by the administration of secret poison. A panic seizes upon society; man *is* afraid "for the terror by night," and for the "arrow that flieth by day," for "the pestilence that walketh in darkness," and for the "sickness that destroyeth in the noonday." He knows not whence the next stroke may come, so unexpected, so unnatural is the source of these crimes; the foundations of all social and domestic confidence are sapped by suspicion, and we think we hear again, as of old, the pathetic lament,—

"*It is not an open enemy that hath done this thing, for then I could have borne it; neither was it mine adversary that did rise up against me, for then peradventure I would have hid myself from him; but it was even thou, my companion, my guide, and mine own familiar friend. We took sweet counsel together, and walked together as friends.*"

It is fearful to think how forcible an illustration of this kind of epidemic is afforded us by the history of the last few months. Crime succeeds crime with unparalleled rapidity, like the monotonous strokes of a moral knell. The editor of the *Tablet* bitterly observes that—

"England is fast becoming a hell upon earth. Pernicious teachings are followed by pernicious practices. Thus the world is horrified within one short month by the harvest of crime which mantles Great Britain with its disastrous and funereal shadow."

More temperately, yet not less forcibly, others set forth the state of society which we have imperfectly depicted. In the *English Churchman* of February 28th, there occurs the following passage, the commencement of a feeling and judicious appeal to the church itself:—

"It is very difficult to refrain from the conclusion that we are, just now, living in the presence of an increased accumulation of greater crimes than has been before witnessed by the present generation. We do not forget the notorious criminals of the first portion of the present half century—the Thurtells and Fauntleroys of that day; but there was not that fearful constellation of crime, as we may term it, which we witness in these days, and which almost every week increases, by some deed which either in the depth of the sin, or the rank of the sinner, shocks and distresses the whole nation. Murders, forgeries, suicides—suicides, forgeries, murders—to say nothing of other sins—have come upon us alternately, with fearful frequency, and in high places as well as low. No sooner had one case spread over the whole kingdom than another occurs to eclipse it, or to dispute a place with it in the public mind. The legislature, commerce, the race-course, the private family, alike contribute to swell the list—the single apartment of the working classes and the stately halls of the aristocracy are equally the scene of 'lamentation, mourning, and woe.'"

The following passage from the *Christian Times* of January 25th enters a little more into the causes of the same phenomena, particularly as to *imitation*:—

"An epidemic of murders seems to be raging just now. We can hardly take up a daily paper without reading of some fresh murder of more than usual atrocity, while the details of the great Rugeley case, dragged slowly to light by the untiring and unerring ministry of science, fill us with horror and amazement that such a series of such crimes should be possible in the broad daylight of our nineteenth century of civilization. . . . . But the Rugeley case is far from being the only one which painfully occupies the attention of the public. During the last weeks, great crimes—especially murders—have succeeded each other with a rapidity which suggests and explains the title of our article. Crime propagates itself by infection, like fever and small-pox, and at times it seems as if the infection came abroad into the atmosphere, and exacted its tribute from every class and every district of the country. The laws of moral infection, and the propagation of moral disorders, are among the most recondite and difficult subjects of contemplation; there is something fearful in the very thought that man may so abdicate his moral freedom as to bring his will and moral nature under the sway of laws as imperious and resistless as those which sustain and balance the orbits of the stars. But we cannot be blind to the fact. There is a large class of minds over which great crimes exert a kind of fascination; and those who have never trained themselves to exercise the responsibilities of moral freedom, are liable to become the victims of the strangest delusions, and catch readily the moral infection which is always lurking, and sometimes raging, in the atmosphere of our world. Let a woman fling herself from the top of the Monument, and the gallery has to be railed in like a wild beast's cage, lest the contagion should spread, and Monument-yard should become the Tyburn of suicides. Let a particular poison have been used with deadly effect in an ignorant and demoralized district, and it must be mixed with some alien substance to colour it, lest it should become the instrument of systematic and wholesale butchery. '*Man that is without understanding is like the beasts that perish,*' said a wise one of old, and in nothing is he more beast-like than in the facility with which he becomes the slave of the laws he was set to govern, and buries his moral freedom literally in the dust."

Whilst writing this very page, a report is put into our hands, of an event which seems from its incredible audacity to put into the shade all those to which allusion is made in these passages. An independent gentleman, resident in one of our largest northern towns, is supposed to have poisoned his young wife with strychnine, actually administered before witnesses, in jelly and other articles of diet; boldly persisted in, in spite of her complaints of their *bitterness*, in spite of others tasting them and confirming her statement. The details are not yet fully known, and we would not prejudge the case; yet the evidence seems so strong and so direct as scarcely to admit of doubt.

The last testimony which it is necessary to adduce as to the actual existence, at this present time, of an epidemic of crime, is part of the address of the Recorder, in the opening of the proceedings of the Central Criminal Court, on March 3rd. It is of great value, as affording legal and official recognition of a most important fact. He thus contrasts the state of England now with its condition two years back:—

"He had before him a return of offences committed down to the year 1854, from which it appeared that, although undoubtedly there was a considerable increase in the amount of crime that had been committed down to that period, yet the increase was mainly in cases of ordinary felony, of a trifling character, and was quite accounted for by the increase in the population and the increased amount of property in the country, and also by the improved condition of the police. As regarded crimes of violence, such as murder, manslaughter, attempts to murder, and other offences of that class, it appeared that during the same period there had been a diminution of such offences to the extent of thirteen per cent. It seemed, however, that it was the same in the history of nations as of individuals, that there were certain periods of great calamities without any apparent traceable cause. During the last twelve-months, after having for forty years enjoyed the blessings of peace, they had been familiarized with all the horrors of war, and there was no doubt that during the same period the most heinous crimes had been committed by persons of high station, by persons also holding a high position in the commercial and banking community, and also by persons in a more humble position of life; and in this court there had certainly been a most unusual number of cases involving the destruction of human life. It was no part of his duty or that of the grand jury to enter into any consideration of the causes that had led to this state of things, nor whether it arose from any peculiar circumstances in the state of the country or of the law; but the subject was one that was entitled to grave reflection, and it certainly ought to urge them all to do everything in their power to extend education among the people, and to improve their condition, as the most effectual means for the prevention of crime."

For the investigation of this lamentable state of society, we propose to make use of the same *calculus* which we have seen to be of such signal service in physical science,—viz., to collect a number of analogous instances, and to analyze the conditions under which they occur, with a view to the ultimate solution of these questions:—

1. What is the condition of mind most calculated for the reception of morbid moral influences?

2. What are the causes and source of this condition?

3. What are the circumstances which *directly* excite and foster these evil tendencies?

4. As a corollary to these,—What are the moral hygienic means to be adopted for the check or prevention of such epidemics?

Were we only to examine the phenomena of disordered *action* in man, we should get but a very imperfect idea of his psychological condition in health and disease. The mind manifests itself by thought, word, and deed; and its disorders are shown* by erroneous ideas, by incoherent discourse, and by unreasonable conduct. These are respectively liable to become epidemic, as in opinion, expression, and crime; and for the complete comprehension of the latter, it is necessary to examine instances of the other two forms. We shall therefore select a few cases illustrative of each, giving the preference to those which have been marked by the most striking psychological phenomena, or which have produced the greatest effects upon the social and political condition of man; only premising, that whilst disordered opinion and action have a much stronger tendency to take on an epidemic type than bodily diseases, their elements *are less complex*, and consequently more susceptible of investigation; a position apparently paradoxical and fanciful, yet one which we believe to be in accordance with experience, and which we hope

* Sir A. Morrison, M.D., on Insanity.

to illustrate afterwards. Many of the most remarkable epidemics, however, are compound, being complicated with physical disorder more or less evident; and these are proportionately *more* complex as to their elements, and present more difficulties to the inquirer than either form taken separately.

Nations, like individuals, have their periods of insanity, excitement, delusion, and recklessness.

"Whole communities suddenly fix their minds upon one object, and go mad in its pursuit; millions of people become simultaneously impressed with one delusion. We see one nation, from its highest to its lowest members, with a fierce desire for military glory; another as suddenly becomes crazed upon a religious scruple; and neither of them recovers its senses until it has shed rivers of blood, and sowed a harvest of groans and tears to be reaped by its posterity."*

Pseudo-religion, opinion practical or speculative, life, property, emotion, all become in turn the subject or the motive for a maniacal epidemic. These collective or *imitative* tendencies appeared very early in the world's history. According to Maimonides, the earth had not been peopled 300 years when *all* turned with one accord to idolatry. Though his account is somewhat fanciful, yet it affords a very probable *theory* of the origin of the class of delusions which, in one form or other, have kept possession of mankind ever since.

"In those days the sons of Adam erred with great error, and the counsel of the wise men became brutish; and their error was this: they said, 'Forasmuch as God hath created these stars and spheres *to govern the world*, and set them on high, it is meet that men should laud and glorify and give them honour.' When this thing was come up into their hearts, they began to build temples unto the stars, and to offer sacrifice unto them, and to worship before them; and this was the root of idolatry. And after this they began to *make images* of the stars, in temples and under trees, and assembled together and worshipped them. And *this thing was spread through all the world;* so in process of time the glorious and fearful Name was forgotten."—Maim. *In Mishn.*

Such was the first origin of idolatry and image worship. After the flood the same tendency was quickly manifested, but under circumstances which indicated a far greater moral perversion and psychical deterioration than before; for this second falling away was especially amongst a chosen people, who had witnessed repeated instances of power which they knew could not reside in wood and stone. "These be thy gods, O Israel," said one, with the bitterest irony, "which brought thee up out of the land of Egypt," pointing to the golden calf which he had been compelled to make. How severely were they satirized by their own pro-

* Mackay's "Popular Delusions."

phets! Idolatry had now taken on its three typical forms—the worship of imaginary powers, of carved images, and of the animate and inanimate objects of nature.

"*I went in and saw; and behold every form of creeping things and abominable beasts, and all the idols of the house of Israel, portrayed upon the wall round about. And there stood before them seventy men of the ancients of the house of Israel, with every man his censer in his hand; and a thick cloud of incense went up. And he brought me to the gate; and behold there sat women weeping for* TAMMUZ (probably Adonis). *And he brought me to the inner court; and behold there were men with their backs to the temple, and their faces toward the east, and they worshipped the sun.*"

The same tendency is indicated in Isaiah's withering sarcasm:

"*He planteth an ash, and the rain doth nourish it. Then shall it be for a man to burn; for he will take thereof and warm himself; yea, he kindleth it and baketh bread; yea, he maketh a god and worshippeth it; he maketh it a graven image and falleth down thereto. He burneth part thereof in the fire; with part thereof he roasteth roast, and is satisfied. And the residue thereof he maketh a god; he falleth down and worshippeth it, and prayeth unto it, and saith, Deliver me, for thou art my god.*"

And to these imaginary deities they sacrificed their sons and their daughters, causing them to pass through the fire. The epidemic of speculative opinion, followed naturally by actual crime, spread over the face of the whole earth; and in this general falling away we find all the elements of the floods of crime which at variable periods since then have well-nigh submerged the moral world. What the condition of the earth was as to general morals and tendencies just before the Christian era, we may indicate by selecting the most refined and civilized of the cities, Rome; and giving the impressions of their own writers, and in their own language, for the vices alluded to are too gross to be completely unveiled:—

"Cum leno accipiat mœchi bona, si capiendi
Jus nullum uxori, doctus spectare lacunar,
Doctus et ad calicem vigilanti stertere naso;
Cum fas esse putet curam sperare cohortis,
Qui bona donavit præsepibus, ———"

And as to the reward of merit, and the mode in which public trust was bestowed:—

"Aude aliquid brevibus Gyaris et carcere dignum,
Si vis esse aliquis; probitas laudatur, et alget."

"— quando uberior vitiorum copia? quando
Major avaritiæ patuit sinus?

But even under this thin veil we may not sully our page with quotations illustrative of the special and universal vices of this vaunted era.

In such a profligate time was Christianity introduced into the world; and for once at least in the world's history the tendency of the human mind to receive opinions *collectively* was directed in a right channel. Promulgated by a few unlettered men—opposed with all the violence of a corrupt priesthood and a pagan court upholding doctrines which human nature felt to be humiliating—persecuted even to the death—Christianity triumphed, and became the religion of the civilized world. But it was not for long that its purity was preserved; errors and heresies crept in; and the doctrines which preached peace on earth and goodwill towards men, were made the pretext for passions the fiercest, persecutions the most diabolical, and wars the most sanguinary that the earth has ever witnessed. There is no wrath and bitterness equal to that which arises in (so-called) religious controversy. Each opinion once promulgated spread like an epidemic, and parties were found to murder each other in support of their respective views, with the more zeal and implacability, the more incomprehensible and less important was the subject of dispute. Ultimately the Christian and the heathen could live without mutual persecution; but the Monothelite and and the Monophysite, the Pelagian and the Arian, ever viewed each other with the most uncompromising hostility.

It would require a large volume even to mention the names of the controversies which for centuries shook the church even to its foundations; we can but briefly allude to a few events, remarkable for their psychical characteristics, their rapid spread, or their bearing upon epidemics of later times.

The Gnostics of the second century originated from the attempt to combine the philosophy of the heathen world with the faith of the Christian. This, as well as the sect of the Manicheans, which arose in the third century, was chiefly remarkable for the incredible rapidity with which it spread, and for its persistency in spite of the severest methods used for its extirpation.

The fourth century is remarkable for the rapid increase of superstition, the reinstitution of *image worship*, the adoration paid to relics, and the many pious frauds, as they have been termed, of the monks. At this time, too, originated that remarkable and long-standing epidemic, which has ever since exercised so powerful an influence over domestic relations and the world generally—that of Monachism. Owing to the prevalence of a certain mystical preaching, vast numbers of men and women withdrew themselves from all society, endeavouring to live by contemplation alone, and mortifying the body by hunger, thirst, and labour. They were gradually reduced to system by Antony, who prescribed rules for their conduct. Some, as the Anachorites, resisted all rule, lived separately, frequented the wildest deserts, fed upon roots, and slept wherever the night overtook them; and all this to avoid the sight of their fellow-creatures. Other sects, as the Sarabaites, were guilty of the most licentious practices, and were indeed profligates of the most abandoned kind.*

The fifth century produced one of the most extraordinary and ridiculous manias that can well be conceived. Simeon, a monk, adopted, as a mark of especial sanctity, the singular device of spending thirty-seven years of his life on the top of a high pillar.

"Seduced by a *false ambition*, and utterly ignorant of true religion, many of the inhabitants of Syria and Palestine followed the example of this fanatic; and what is almost incredible, this practice continued in vogue till the twelfth century."—Mosheim, *Eccles. Hist.*

The rise and spread of Mahometanism in the seventh century is one of the most remarkable instances of the rapid propagation of ideas and principles. Doubtless the terror of Mahomet's arms, and his repeated victories, were very irresistible arguments; but at the same time his law was wonderfully adapted to the corrupt nature of man; its requirements were few and easy, its articles of faith simple, and its promised rewards marvellously acceptable to the manners and customs of the Eastern nations, and their favourite vices. "It is to be observed," says Mosheim, "further, that the gross ignorance under which the Arabians, Syrians, and Persians, and the greater part of the Eastern nations, laboured at this time, rendered many an easy prey to the artifice and eloquence of this bold adventurer." When we add to this the dissensions and animosities amongst the Greeks, Nestorians, and others, which filled the East with carnage, assassinations, and other enormities, such as made the very name of Christianity detestable, we may cease to wonder at the spread of of any new religion. Will not an attentive consideration of these reasons in the aggregate suggest to the reflective mind the source of some of the remarkable heresies of the present day, as our Mormonism and Socialism, and the German Apostolico-Baptism?—The epidemic of the eighth century was a violent contest, which overspread the whole Christian world, between

* Trench, in his "Lectures on Words," has some remarks on this point of great interest.

"This is a notable example of the manner in which moral contagion, spreading from heart and manners, invades the popular language in the use, or rather misuse, of the word 'religion.' In these times, 'a religious person' did not mean one who felt and allowed the bonds that bound him to God and his fellows, but one who had taken peculiar vows upon him. A 'religious' house did not mean in the Church of Rome a Christian household ordered in the fear of God, but a house in which persons were gathered together according to the rule of some man. What an awful light does this one word, so used, throw upon the entire state of mind and habits of thought in those ages!"

the Iconoduli and the Iconoclastæ, concerning image worship, as their names imply. The ninth century presents to us the origin of the trials of innocence, which for ages continued so popular—by water, by single combat, by the fire ordeal, and by the cross.

The first is of great interest, as being afterwards so universally made use of, in the detection of supposed witches. The person suspected of any crime was thrown into water, the right hand bound to the left foot; if he sank, he was esteemed innocent; if he floated, it was evidence of guilt. In the trial by duel, the survivor was considered to have proved his innocence. In the fire-ordeal, the accused person walked barefoot on heated ploughshares, or held a ball of red-hot iron in his hand; if innocent, these feats would be accomplished without injury. In the last form of trial, that by the cross, the contending parties were made to stretch out their arms, and he that could continue in this posture the longest gained his cause.* The universal belief in the infallibility of these tests is not the least singular feature in the mental aspect of these ages.

In the tenth century a strange panic seized upon men's minds, and produced the most disastrous effects. They conceived that the end of the world was close at hand, and vast multitudes forsook all their civil and domestic ties, gave their property to the church, and repaired to Palestine, where they imagined they should be safer than elsewhere. An eclipse of the sun or moon was considered as the immediate precursor of the end of all things; the cities were forsaken, and the wretched inhabitants did actually hide themselves in caves and rocks. Others attempted to bribe the Deity, by great gifts to the church; others pulled down palaces and temples, saying that they were of no more use. "In a word, no language is sufficient to express the confusion and despair that tormented the minds of miserable mortals on this occasion."

Consequent upon this was perhaps the most extraordinary epidemic into which fanaticism ever ran. We have said that vast multitudes left their homes to go to the Holy Land: not a meteor fell across the sky, but sent whole hordes on the same delusive errand. The hardships they suffered on the way were almost incredible; yet they were exceeded by those experienced from the Turks when they reached their destination. Persecution of every kind awaited them; they were plundered and beaten, and not allowed in most instances to enter Jerusalem. By degrees, this particular epidemic dread began to subside, and some of these pilgrims returned to Europe full of the indignities which they had received. Amongst them was an enthusiastic and eloquent, perhaps half crazy monk, Peter the Hermit, who,

* A different account of the test of the cross is given by many writers, but this appears to have been the original one.

on his return, convulsed Europe by his preaching and his story of their wrongs. Then resulted a scene such as the world had never witnessed. In the insane idea of wresting Palestine from the Turks, countless myriads of fanatics left their homes, and traversed Europe under circumstances unparalleled in the history of man. Why should we dwell upon the details of the Crusades? Hundreds of thousands perished on the way; the roads and fields were heaped up with corpses; the rivers were dyed for miles with their blood. Yet again and again schemes for the accomplishment of the same purpose were adopted: now the elements were the lowest and vilest of the people—now the flower of Europe's chivalry,—and again thousands of *children* formed a separate crusade of their own. Millions of treasure were expended, and two millions of lives sacrificed, in the two hundred years during which this disastrous moral epidemic prevailed. And *this* ended!—the philanthropist would fain hope that such a fearful convulsion would not pass without some purification of the atmosphere.

Scarcely had the excitement of Europe subsided when another scourge made its appearance. The great Plague, or Black Death, of the fourteenth century, appeared in 1333 in China, and passing over Asia westward, and over Europe and Africa, carried off about one-fourth of the people. In Europe alone it is supposed that twenty-five millions fell victims to this fearful pestilence.

All epidemic diseases have their *moral* aspect; and this one was attended by a constellation of fanaticisms and delusions such as man has never witnessed before or since. The belief in witchcraft was already very prevalent, and there had been some isolated persecutions directed towards it. But the specific moral aberrations connected with this period were:—

1. The rise and spread of the Flagellants, or Whippers.
2. The wholesale murder of the Jews, on the suspicion of having poisoned the water.
3. The dancing mania.

The compound aspect of these three has more than an ordinary interest to the philosophic mind, arising from the fact, that although the first two appear to be of a strictly *psychical* nature, a *somatic* origin is indicated, from their extremely close connexion with the latter, which was accompanied by the most striking and uniform physical derangements, very analogous to the phenomena of hysteria. The sect of the dancers, indeed, seems to serve as a connecting link between mental and bodily affections, and to lead by a natural transition to many of the convulsive forms of religious worship with which the present century is familiar, as the Jumpers, the Shakers, the preaching mania in Sweden in 1842, &c.

The primary notion of the "Whippers" may be traced to the fact, that for ages flagellation had been considered by the Church the most appropriate punishment and atonement for vice. Horrified by the ravages of the plague, in deadly terror of its advances, the people thought to stop the vengeance (as it was supposed) of Heaven by mortifications and penance. Almost simultaneously, in many parts of Hungary and Germany, large masses of the lowest orders of the people formed themselves into bodies, which marched in procession through the cities, robed in sombre apparel, covered with red crosses, bearing triple knotted scourges, in which points of iron were fixed.

"It was not merely some individual parts of the country which fostered them: all Germany, Hungary, Poland, Bohemia, Silesia, and Flanders did homage to the mania. The influence of this fanaticism was great and threatening; resembling the excitement which called all the inhabitants of Europe into the Deserts of Syria and Palestine 250 years before."—Hecker.

They performed penance twice a day, scourging themselves and each other till the blood streamed from them; and this they blasphemously said was mixed with the blood of the Saviour. Flagellation was held to be superior to, and to supersede all other observances; the priests were forsaken, and these "Brethren of the Cross" absolved each other, and took possession of the churches, where their enthusiastic songs affected greatly the minds of the people.

As might be expected, all this speedily resolved itself into licentiousness and crime. The Church and the secular arm combined to put a stop to this universal phrenzy; veneration turned, in many places, into persecution, and public burnings of the chief instigators of the riots became common. During this and the early part of the next century a constant contest was carried on with them, but the sect was found most difficult to eradicate.

Simultaneously with these proceedings, was instituted a bloody and barbarous persecution of the Jews. Amongst the other absurd conjectures as to the source of the terrible pestilence which was everywhere raging, the Jews were supposed to have poisoned the wells or infected the air. They were pursued with relentless cruelty—tortured, in many instances, into a confession of crimes which had never been committed, and then burnt alive.

"Whenever they were not burnt, they were at least banished; and so, being compelled to wander about, they fell into the hands of the country people, who, without humanity, persecuted them with fire and sword."—Hecker's *Epid.*

In some places, driven to desperation, the Jews fired their own quarter of the town, and so perished. At Strasbourg, 2000

were burnt alive in their own burial-ground. In Mayence, it is supposed that 12,000 Jews were slaughtered by the Flagellants.

"At Eslingen, the whole Jewish community burned themselves in their synagogue; and mothers were seen throwing their own children on the pile, to prevent their being baptized, and then precipitating themselves into the flames."—Hecker.

A singular feature presents itself in the progress of this epidemic persecution, as in that of witches, to be shortly noticed,—viz., that after the rage had lasted some time, many confessed voluntarily, and without torture, to the crimes of which their countrymen were accused; and it even appears probable that some actually attempted to commit them by putting certain poisons into the waters. Apparently an irresistible impulse leads to acts of this nature, from the constant dwelling of the mind upon the accusations and reports on the subject. We meet with analogous instances in all epidemics of crime, and it is not unfrequent to meet with those who, from a morbid desire for notoriety, will insist upon confessing crimes which have evidently not been perpetrated, such as the murder of people still living.

The humanity and good sense of Clement VI. at last succeeded in putting a stop to this wholesale butchery; but it was not till after scores of thousands had fallen victims to the insane and cruel delusion.

The *Dancing Mania* next claims attention. In his preface to an account of this affection, Hecker makes some interesting reflections, which we here quote:—

"These diseases afford a deep insight into the workings of the human mind in a state of society; they expose a vulnerable part of man,—the *instinct of imitation*,—and are therefore very nearly connected with human life in the aggregate. It appeared worth while to describe diseases which are propagated on the beams of light, on the wings of thought, which convulse the mind by the excitement of the senses, and wonderfully affect the nerves, the media of its will and feelings. It seemed worth while to attempt to place these disorders between the epidemics of a less refined origin, which affect the body more than the soul, and all those passions and emotions which border on the vast domain of disease, ready at every moment to pass the boundary."

About 1374, at Aix-la-Chapelle, the singular spectacle was presented, of groups of men and women, who would join hands, forming a circle, and dance for hours together in wild delirium, till they fell to the ground utterly exhausted.

"They then (says Hecker) complained of extreme oppression, and groaned as if in the agonies of death, until they were swathed in cloths bound tightly round their waists,—a practice resorted to on account of the tympany which followed these spasmodic ravings; but the bystanders frequently relieved patients in a less artificial manner, by thumping and trampling upon the parts affected."

It seems that in this and the analogous affections,—the preaching mania in Sweden, the convulsive disorders in Shetland, and the *convulsionnaires* in France,—the most brutally violent means were adopted for the removal of this tympany, not only without pain to the sufferer, but with actual temporary relief. Referring to this last class, M. Littré says:—

"Ni les distensions ou les pressions à l'aide d'hommes vigoureux, ni les supplices de l'estrapade, ni les coups portés avec des barres ou des instruments lourds et contondans, n'étaient capables de léser, de meurtrir, d'estropier les victimes volontaires." *

Many called out for heavy weights to be thrown upon them, and for the blows to be administered with more force upon the abdomen. A stone about thirty pounds in weight, called *a pebble*, was in frequent use for this purpose.

This dancing mania rapidly spread over the Netherlands, which were overrun with troops of half-naked dancers.

"At length the increasing number of the affected attracted no less anxiety than the attention that was paid to them. They took posesssion of the religious houses, processions were instituted, masses were said for them, and the disease—of the demoniac origin of which no one entertained the least doubt—excited everywhere astonishment and horror. They were much irritated at the sight of red colours, the influence of which on the disordered nerves might lead us to imagine an extraordinary accordance between this spasmodic malady and the condition of infuriated animals."—Hecker, p. 89.

In this, as in all other epidemics, opportunity was found for the wildest licentiousness; gross impostures mixed with the real disease, and ultimately the resultant vices excited the indignation of clergy and laity, who united to put a stop to the disorders.

"Meantime, the plague crept on, and found abundant food in the tone of thought which prevailed in the fourteenth and fifteenth centuries, causing a permanent disorder of the mind, and exhibiting, in those cities to whose inhabitants it was a novelty, scenes as strange as they were detestable."—Hecker.

Nothing affords a more striking illustration of the tendency which opinion, emotion, and action have, to take on a *collective* aspect, than the subject of WITCHCRAFT, whether considered as to its millions of votaries, its tens of thousands of persecutors, its myriads of victims, or the curious psychological phenomena developed by the mutual reactions of these.

* *Rev. des Deux Mondes*, February 15, 1856.

"Europe for two centuries and a half brooded upon the idea, not only that parted spirits walked the earth to meddle in the affairs of men, but that men had power to summon evil spirits to their aid to work woe upon their fellows. An epidemic terror seized upon the nations; no man thought himself secure, either in his person or his possessions, from the machinations of the devil and his agents. Every calamity that befel him he attributed to a witch. France, Italy, Germany, England, Scotland, and the far North, successively ran mad upon this subject—thousands upon thousands of unhappy persons fell victims to this cruel and absurd delusion."—Mackay's *Popular Delusions*.

The summary of belief was something to this effect. At the command of any one who would sell his soul, in exchange for certain services during a stated period, there were innumerable demons—Wierus says only 7,405,926—incubi and succubi, that is, male and female, taking on various forms, according to the circumstances required—but if *human*, always imperfect in some respect.

They were bound to obey any order, except to do good, in which case they disobeyed, and visited their displeasure upon the offender. At uncertain intervals—generally on the Friday night—there were meetings, called the "Sabbath," at which those who in the intervals had done sufficient evil were rewarded, and those who had not, received chastisement from Satan himself, who flogged them till they could neither sit nor stand. New-comers were admitted by the ceremony of denying their salvation, spitting upon the Bible, and vowing obedience to "the master." Their amusement on these occasions was a dance of toads—their banquet, things too disgusting to mention. A general examination was made to know if each possessed "the mark," by which they were recognised as the "Devil's own." This mark was insensible to pain. Those who had it not, then received it. When the cock crew, the Sabbath ended, and all disappeared.

The persecutions on account of witchcraft were carried on from various motives—political, as in the extermination of the Stedinger in 1234, by Frederic II., assisted by the Duke of Brabant and others; and in that of the Templars, accused of sorcery by Philip IV. of France and burned; religious, as in the persecutions of the Waldenses under this pretext; and superstitious, as in the innumerable trials for witchcraft with which the middle ages abound. It is computed that during the prevalence of this epidemic, at least one hundred thousand persons were burnt as witches or sorcerers.

Illustrative of the strange psychological phenomena manifested in the votaries of this belief, and their *collective* character, we quote some facts and observations from a profoundly philosophic article in the *Revue des Deux Mondes* for February 15th, by

M. Littré. It will save repetition to remark, first, that there is a singular uniformity in the confessions of those accused; second, that although many confessions were elicited by torture, and many made through *dread* of torture, yet due allowance made for all these, there remain many who confessed *voluntarily*, and manifested pride in their supposed powers; speaking with delight of their enjoyments at "the Sabbath," and longing to be burned that they might constantly enjoy "the master's society."

Under the pontificate of Julius II., many thousands of persons were burnt, who confessed freely, that in the form of cats they were in the constant habit of destroying children.* M. Littré on this makes the following striking observations:—

"In this fact, for which during many years the pile was constantly erected, we remark at first one prominent phenomenon, *i.e.*, its *collective character*. All the sorcerers say that they were changed into cats; and this in spite of the punishment which awaits them; they accuse themselves of homicides without number. In confirmation, the mothers notice spots of blood on the dead children; the fathers speak of strangely pertinacious cats about the house. To all this tragedy, so well attested on all parts—sealed by confession, certified by solemn inquisition—there fails but one thing: *in spite of the assassinations of so many children, the mortality is not increased, nor the district depopulated.*"

In the sixteenth century, the nuns of a certain convent were all seized with a kind of hysterical affection. Naturally they were bewitched, and victims had to be burned before they were cured.

In Lorraine, from 1580 to 1595, about nine hundred persons were burnt on this pretext. They all saw the Devil near them, even whilst the torture was being inflicted, endeavouring, in his way, to comfort them. In Labourd, about the beginning of the seventeenth century, the confessions of the accused are still more remarkable:—

"La plupart parlaient avec une expression passionnée des sensations éprouvées au Sabbat; ils peignaient en termes licencieux leur enivrement; beaucoup déclaraient être présentement trop bien habitués à la société du diable pour redouter les tourments d'enfer; souffrant fort joyeusement qu'on leur fit leur procès, tant elles avaient hâte d'être avec le diable; elles s'impatientaient de témoigner combien elles dé-

* The witch *mania* may be considered to have first fairly set in in 1488, when Pope Innocent VIII. launched his terrible manifesto against them. In this celebrated bull he called upon all the princes of Europe to assist in extirpating this crime, by means of which all manner of wickedness was wrought. He also appointed inquisitors in every country, armed with little less than apostolic power, to try and punish the accused. Naturally this crusade against a supposed crime propagated it, and wonderfully deepened the belief in the minds of the people.

siraient souffrir pour lui, et elles trouvaient fort étrange qu'une chose si agréable fut puni."

It is unnecessary further to multiply instances; we have said enough to illustrate the eminently *collective* character of these phenomena—"seizing upon great numbers simultaneously, and subjugating them to the same class of sensations and actions, finally passing away, and leaving no trace, save the remembrance of their singularity, and the difficulty of theorizing upon them."

We can scarcely persuade ourselves that some of the so-designated "*spiritual*" manifestations of our own times are less absurd or dangerous than those just quoted. In concluding this branch of the subject, one or two general observations suggest themselves which are both of speculative and practical interest.

1. The immense number of convictions and executions for witchcraft are easily accounted for, when we consider the rules and tests for the detection of the supposed crime. These, it is well known, were so devised as to reflect no discredit on the accuser in case of failure, but to admit no loophole of escape for the accused.

2. In addition to the surprising uniformity of the confessions, there is another evidence of the strength and persistency of the delusion. When the mania for witch-extermination had begun to subside, and men were more anxious to acquit than condemn, there were found numbers who voluntarily accused themselves of crimes evidently not committed, as of the murder of people still living, and of having attended at the "Sabbath" during nights when the strictest watch had been kept upon them, and it was evident they had never quitted their room.

3, and lastly. The most remarkable consideration of all is this —and it shows forcibly the inconsequence of the whole business. These people, who could raise tempests, who partook of the power of the Prince of Darkness, who could work their will amongst the elements—*they* had neither riches, nor power, nor grandeur; they, who could change their form at pleasure, could ride through the air, and pass through keyholes and crevices, and up chimneys at will—these very people could not preserve themselves from a painful and ignominious death!

The epidemics of the sixteenth, seventeenth, and eighteenth centuries we can but name in passing. The sixteenth was eminently *reformative*, and never, not even in the sixth and seventh centuries, did polemic rage burn more hotly than during this period: the specific fanaticism was that of the Anabaptists, who, under the pretext of zeal, kept Europe in an uproar.

The seventeenth century in England and the eighteenth in France present striking analogies to each other in their broad features of resistance to authority. In each case, the entire national mind, in all its manifestations, thought, expression, emotion, and action, was disturbed to the very foundation. In each there was a period of luxuriant literature, followed by deep thought amongst the masses. In each, prolonged thought excited emotion; and this in its turn produced action, reaction, violence, anarchy, *despotism*. In each case, after peace was restored, there was another phase of literature, remarkable for its immorality.* The eighteenth century also presented two of the most frantic commercial manias that the world has ever witnessed—in France, Law's Bank and Missisippi scheme; in England, the South Sea scheme. It is impossible even to glance at the nature of these projects, or to describe the excitement caused by their rise and progress—the desperation and ruin consequent upon their failure. They were instituted in the same year; the two nations went mad simultaneously; and in the same year, 1721, both broke down, reducing thousands of families to beggary. Each gave rise to innumerable other bubbles, none of which were too absurd to be adopted. At one time, eighty-six of these undertakings were declared illegal by the Committee of the House of Commons, and abolished accordingly. Number seventeen in this list will serve as a fair sample of the credulity of the period. It was entitled: "A company for carrying on an undertaking of great advantage, *but nobody to know what it is!*" The projector of this cleared 2000*l*. in five hours, and decamped.

When remarking upon the mental aberrations of our own century, the nineteenth, M. Emile Montegut observes:

"Ils n'ont plus le fanatisme révolutionnaire de leurs pères, et ce n'est pas eux qui demanderaient *à étrangler le dernier roi avec les entrailles du dernier prêtre!*"

True—and fortunate as true—our tendencies are not so rabid; yet we take our part bravely in the insanities of our race. There are few of the manias which have been already noticed that have not their representatives in the present age. Penance, mortifications, and dancing—panic-terror, witchcraft, and commercial speculation run wild—a revolutionary madness pervading an entire continent,—we seem to be taking a *résumé* of the world's follies and crimes. But one morbid tendency stands out in bold relief from the rest—that of spiritualistic fanaticism, as set forth by jumpers, shakers, apostle-baptists, socialists, mormons, spirit-rappers, and a crowd of other sects, each claiming exclusive possession of the truth. Each one might well require a volume to relate their history and doings. We will but briefly notice

* For an account of the causes of this state of literature in England, see Macaulay's "Hist. of Eng." vol. i. p. 399, *et seq.* The corresponding condition in France is alluded to in Alison's "Hist. of Europe from 1815 to 1852," vol. v. p. 274.

two, which are remarkable for the strange social and civil effects produced by them upon our transatlantic brethren.

Joseph Smith, the inventor of mormonism, which has now its tens of thousands of votaries encamped in the valley of the Salt Lake, was a man from amongst the lowest of the people. His character is naively described by M. Montegut, as not possessing precisely the innocence of a virgin! According to the same authority, he was of licentious manners, an audacious liar, a bankrupt, an adulterer, a murderer. The following passage would lose by translation, and affords matter for profound thought:

"Eh quoi! peut dire un sceptique, voilà un homme notoirement connu pour le dernier des mécréans et des coquins; un homme d'une éducation vicieuse, d'une intelligence médiocre, d'une âme rapace, et grossièrement sensuelle; un homme qui se recommande simplement par un appetit solide, un front d'airain, des doigts crochus et agiles; cet homme réussit, non pas à voler une compagnie d'actionnaires ou à inventer un moyen subtil d'ouvrir les serrures, mais à fonder une religion, et à entraîner sur ses pas de grandes multitudes qui révèrent son nom! Il publie une fausse Bible, on l'accepte pour vraie: il se donne pour le prophète de Dieu, et il le fait croire sans trop de difficulté; il établit des dogmes qui blessent tous les sentiments de liberté des Américains, et il trouve des Américains pour accepter ses dogmes; il proclame la déchéance de la femme dans un pays où elle est plus véritablement souveraine que dans aucune contrée de l'Europe, et il se rencontre des femmes pour venir se remettre entre ses mains!"*

Add to this, that, professing to live in such sanctity and close communion with God as to be able to raise the dead, his life was one of the most open profligacy, with details too sickening to mention, and that his followers are numbered by myriads,—and we have a sufficiently curious yet melancholy example of the credulity of large masses. The religion professed is eminently *eclectic;* each previous one contributing that part which is most acceptable to the appetites and passions of man. We cannot enter further into detail; sufficient has been said to vindicate the *collective* character of this delusion.

The next epidemic which we have to notice is still more extraordinary in its psychological relations, and forms an appropriate climax to this part of our sketch.

The Spirit Faith in America is computed to embrace two millions of believers, and hundreds of thousands in other lands, with 20,000 mediums. It appears that these include men in all ranks of society, from the highest to the lowest. Many of the facts related imperatively demand that we should consider this as a *delusion,* not altogether an *imposture,* especially the consideration

* *Rev. des Deux Mondes,* Fév. 15.

of the number who have gone insane on the subject. It is said that amongst the lunatics confined in public asylums in the United States, there are 7520 who who have become such entirely owing to this "spirit faith." The spiritualist has no *fixed* creed, but finds his "articles" as he advances. The fundamental belief is in their communication with disembodied spirits through the means of *mediums*—persons who are sensible of the presence of these spirits, and can learn and interpret their will. There are "rapping mediums," whose mode of action is sufficiently well known; there are the "writing mediums," who in a kind of cataleptic trance write down the communications of the spirits. There are also the "speaking mediums." On these last, M. Littré has the following remarks:—

"Ceux-ci sont des véritables pythonesses; d'une voix souvent différente de la leur, ils prononcent des paroles qui leur sont inspirées, ou qui sont mises directement dans leur bouche. Cette passiveté a été notée chez les convulsionnaires. Plusieurs parlaient comme si les lèvres, la langue, tous les organes de la prononciation eussent été remués et mis en action par une force étrangère; dans l'abondance de leur éloquence, ils leur semblaient qu'ils débitaient des idées qui ne leur appartenaient aucunement, et dont ils n'acquéraient la connaissance qu'au moment où leurs oreilles étaient frappées par le son des mots. Une des prophétesses disait, et ce qu'elle déclarait s'appliquait à des milliers d'autres—'Je sens que l'esprit divin forme dans ma bouche les paroles qu'il me veut faire prononcer. Pendant que jeparle, mon esprit fait attention à ce que ma bouche prononce, comme si c'était un discours récité par un autre.'"

Interpreted by these three orders of *media*, the spirits give information on all subjects upon which they are consulted,—religious, social, political, or medical. They relate past events, interpret present ones, and prophesy the future. It would appear, however, that the spirits have not all the wisdom popularly attributed to "ghosts," for they make frequent mistakes both as to past and present, whilst their knowledge of the future is dealt out economically and oracularly. Their religious instructions are involved in a vague mysticism, and their social, domestic, and political directions would, if followed, often lead to remediless confusion. It is, nevertheless, a thriving trade, for the revelations of the invisible world are made a matter of merchandise, and as publicly advertised as any other quack medicine!

These phenomena are closely allied, on the one hand, with those of trance and hysteria, and on the other with those of witchcraft and demoniacal possession, of the prophecies of Cevennes, the "preachings" of Sweden, the apostle-baptists of Germany, and the convulsionaries of St. Médard.

M. Littré suggests an ingenious theory of their *somatic* origin; which we shall endeavour to condense. He entirely disbelieves, in the outset, in their spiritual origin,—first, from the smallness and absurdity of the results produced; secondly, because all the manifestations are such as, in a *sporadic* form, are well known, and recognised as the normal symptoms of certain pathological conditions of the nervous centres.

These phenomena are all resolvable into disorders of the senses, muscular actions, and intelligence; and M. Littré shows first how these may all be affected by well-known physical agents, producing certain definite physiological results. Thus illusions of the eye may be produced by belladonna,—those of the ear by large doses of quinine. The muscular system may be convulsively affected by the administration of strychnine, whilst a general modification (or even aberration) of the intelligence and the emotions, is producible at will, by the use of opium, hachish, and other narcotics.

These results are all physical,—they are likewise all special, definite, and constant. Whence it may be considered as ascertained,—

(1) *That a certain physiological (or pathological) condition of the nervous centres is connected with illusions.*

But (2) it is well known that whatever subjective sensations may be produced by *external* agency may also be produced by *internal* changes,—*i. e.*, changes in the organs themselves. Thus, from congestion and other causes, the eye may perceive light, the ear may perceive sound, without those being actually present; and so with the other senses. Under similar circumstances, the intelligence is troubled, creates strange associations of ideas, sees visions, and appears abstracted from a real world to live in an imaginary one. Here we have the same condition as that referred to above, produced spontaneously—yet the source is somatic or physical.

But (3) we know that certain pathological conditions have a tendency to become epidemic, influenced by causes not yet investigated, as glandular, bronchial, and gastric inflammation or irritation, in time of plague, influenza, or cholera; and it is not unreasonable to conjecture that the morbid change in the nervous centres, which we see in *individual* cases producing such visionary results, may also become epidemic, and produce these aggregate delusions.

On reviewing the foregoing details, we see how strong is the tendency of opinion once promulgated to run into an epidemic form,—no opinion, no delusion is too absurd to take on this collective character. We observe also how remarkably the same ideas reproduce themselves, and re-appear in successive ages. We have now to examine those cases in which *individual* crime operates upon masses of people to produce great numbers of imitations. We shall see that no crime is too horrible to become popular,—homicide, infanticide, suicide, poisoning, or any other diabolical human conception.

Crime of various kinds appears to be *endemic* in certain countries, and even to be incorporated in the forms of religion peculiar to them. Assassination was one of the principal *observances* among the subjects of the Old Man of the Mountain, a sect which lasted nearly two centuries, and carried dismay and terror into every Court in Europe. Infanticide is a part of the religion of the Hindoos. It is stated in Buchanan's Researches in Asia, that the number of infants killed in one year in the two provinces of Cutch and Guzerat was 30,000. It is also endemic in China; the number of children exposed in Pekin alone is about 9000 annually. It is much the same in the South Sea Islands, the Sandwich Islands, and Ceylon. Suicide appears to be endemic in Hindostan; many hundreds lay violent hands on themselves each year—three-fourths being women. Robbery is endemic in Italy; incendiarism and murder, we regret to think, in Ireland. But though these seem to be the favoured *habitats* of the special crimes mentioned, yet *everywhere* are the seeds of evil sown deep under the surface of society, deep in the corrupt moral nature of man, and their development is like those curious phenomena so familiar to the observer of animal life in its most elementary forms; where it only is required that the proper nidus should be prepared, and countless millions of living creatures crowd in, or originate from it, propagating themselves with ever geometrically increasing rapidity—the germ ever present—the *conditions* casually supplied.

So let the surface of society be disturbed, or its depths ploughed up by influences of exceptional social, commercial, or political events, as in times of speculation, panic, or war, then inevitably will these seeds of evil works germinate, and their results will be offences against order, property, and life, which for their check will often require enactments as stern and unsparing as the fiat by which the thistle and the poppy are eradicated from our corn-fields. In epidemics of plague, cholera, or influenza, we can trace those conditions of public hygiene which are calculated to favour or retard their development; but the *cause* of the rapid spread at that particular period remains a mystery. We believe that the causes of the spread of crime are *more* amenable to investigation than these; that the imitative propensity, so closely bound up with the constitution of man, his impulses, weaknesses, and vices, taken in combination with the special, social, or political conditions of any given time, are amply sufficient to account for our natural principles, and to reduce to some sort of law these striking *collective* moral aberrations. We proceed to give a few illustrations of these aggregates of crime, with a view to an inquiry into the causes concerned in their production: (1) as to crime against property, (2) against person and life.

Mr. Macaulay gives a very graphic picture of an epidemic of housebreaking and robbery, in the fourth volume of his recent History. After alluding to the scarcity of grain, he says:—

"A symptom of public distress much more alarming was the increase of crime. During the autumn of 1692 and the following winter, the capital was kept in constant terror by housebreakers."

Attempts were made on the mansion of the Duke of Ormond and the Palace at Lambeth.

"From Bow to Hyde Park, from Thames-street to Bloomsbury, there was no parish in which some quiet dwelling had not been sacked by burglars. Meanwhile the great roads were made almost impassable by freebooters, who formed themselves into troops larger than had ever been seen. The Oxford stage-coach was pillaged in broad day, after a bloody fight. A wagon laden with 15,000*l.* of public money was stopped and ransacked. The Portsmouth mail was robbed twice in one week, by men well armed and mounted. Some jovial Essex squires, while riding after a hare, were themselves chased and run down by nine hunters of a different sort, and were heartily glad to find themselves at home again, though with empty pockets."

It seems that these robbers were by some suspected of being Jacobites; but they showed the most laudable impartiality in the exercise of their calling. The gang, consisting of not less than eighty names, were ultimately betrayed by the confession of one of their fraternity.

Another form of crime against property is that of Incendiarism. History abounds with instances of this offence. We shall but mention two cases, which will illustrate the mode in which the propensity is propagated. M. Marc, in his "Annales d'Hygiène Publique," relates some particulars of a band of incendiaries, who in 1830 (the date is significant) desolated many departments of France. A girl, about seventeen years of age, was arrested on suspicion of being connected with them. She confessed that "twice she had set fire to dwellings by instinct, by *irresistible necessity*,—a victim to the suggestions to which she was exposed by the constant reports of fires, and the alarms from these scenes, which terrified the whole country and excited her diseased brain." A boy, about eighteen, committed many acts of this nature. He was not moved by any passion; but the bursting out of the flames excited a profoundly pleasing emotion, which was augmented by the sound of the alarm bells, the lamentations, clamours, and disorders of the people. "Dès que le son des cloches annoncait l'explosion de l'incendie, il était forcé de quitter son travail, tant son corps et son esprit étaient violemment agités."

In all this we find nothing mysterious, though the epidemic is strongly developed. A time of political excitement and change (1830)—men's minds agitated—revenge for real or supposed injuries influencing the few—imitation and impulse inducing the many to follow—hysterical girls—excitable and idle boys (for most of the band were young)—we have here a sufficient number of elements well known to exist, and ready to burst forth into crime when the example is once set, and quite capable of themselves producing the entire phenomenon.

In De Quincey's curious and brilliant paper entitled "Murder considered as one of the fine arts," he observes, with regard to this class of crime, that "it never rains but it pours," and gives some singular illustrations of its tendency to occur in groups. He mentions that in the comparatively short time intervening between 1588 and 1635, seven murders or assassinations of the most distinguished characters of the time occurred. The first was that of William I. of Orange; then Henry Duke of Guise; next to him, Henry III., the last of the Valois princes; next, Henry IV., the first of the Bourbon dynasty. Then followed the murder of the Duke of Buckingham, of Gustavus Adolphus, and lastly of Wallenstein. It is not often in the history of man that such a constellation of crime is met with; yet epidemics numerically more formidable are constantly presenting themselves. One murder of great atrocity is constantly and (as it would appear) inevitably followed by others vieing with it in horror. Sometimes, also, a predominant delusion affecting large numbers gives rise to many examples of the same crime. Thus in Denmark, in the middle of the last century, a great number of people were affected with the morbid notion, that by committing premeditated murder, and being afterwards condemned to die, they would, by public marks of repentance and conversion on their way to the scaffold, be better prepared for heaven. The murders were generally committed on children.

As it was evident that capital punishment would not stop this epidemic, it was ordered that the delinquents should be branded on the forehead, confined for life to hard labour, and annually publicly whipped. A midwife in Paris for some time was in the habit of introducing an acupuncture needle into the brains of new-born children, that they might people heaven!

Esquirol relates a curious case of homicidal monomania, which created much excitement. He was within a short time called in to many others, all of whom traced the tendency to this original case:—

"Un monsieur lit un journal dans lequel sont rapportés les détails du meurtre d'un enfant; la nuit suivante, il est éveillé en sursaut avec le désir de tuer sa femme. Une femme coupe la tête à un enfant qu'elle connaissait à peine, est traduite en jugement; ce procès a beaucoup de retentissement, et produit par imitation un grand nombre de monomanies homicides."

The acquittal of Oxford for shooting at the Queen was quickly followed by the attempt of Francis to imitate him. The case of Laurence, who in 1844 killed an inspector of police, was immediately followed by that of Touchett, who, without motive, save that of imitation, shot a stranger at the shooting-gallery. A similar instance of succession, with its causes, is alluded to in the following paragraph, extracted from the *Medical Times*:—

"It is known that Mallard, the pawnbroker from whom Wix purchased the pistol with which he shot Bostock, his master, was the shopkeeper from whom Graham subsequently bought the pistol with which he shot the stranger, Blewitt. This fact, sufficiently striking of itself, is made more remarkable by the pawnbroker's evidence, which tends to prove that what looks like a mere coincidence was, in fact, but the operation of a moral law, and that where the appearance was an accident, the reality was a principle. '*Immediately*,' says the pawnbroker, '*after the assassination by Wix, I received a great many applications for pistols, and now, within the last few days*' (after the second tragedy) '*several persons have applied to me for the same thing.* I am now determined, however, never to sell another.' Passing by the very proper resolve adopted by this tradesman of mishaps, we find in the fact he records, a startling revelation of the mental condition of a portion of that public authors and orators are so fond of bepraising. To many of our London denizens there would appear to exist a fascination about the circumstance of murder. About us and near us, arrayed in all the externals of common sense and charity, are persons endued with a mesmeric sensitiveness to the horrors of homicide, from the very intensity of whose abhorrence of crime arises an interest for it, tempting and fascinating them to its commission."

The homicidal horrors of the French Revolution partook strongly of the nature of an epidemic. Here everything cooperated to propagate the slaughterous tendency: times when political changes were almost of daily occurrence; distress amongst the people; gradual loss of respect for human life in general; self-defence, terror, emulation; morbid imitation; mere sanguinary impulse;—all were in operation to produce scenes such as man had never before witnessed.*

In general, when unconnected with national interests, the mere homicidal epidemic must, for obvious reasons, be comparatively

* It is interesting to trace in these cases the effect of any physical agent, however unable we may be to comprehend its *modus operandi*. Esquirol says: "Lorsque le terrible klamsin souffle, l'Indien, armé du fer homicide, se précipite sur tout ce qu'il rencontre." Similar to this is the "running amuck" of the Malay, when drunk with bang, hachish, or enthusiasm.

limited in its extent. There are other forms, however, not less criminal, where the same restrictive causes are not in operation: the only one we shall at present notice is the crime of duelling. In the year 1528, Francis I. sent a cartel to the Emperor, Charles V.; and from this time the duel became a fashionable vice—very shortly after amounting to an epidemic. In the reign of Henry IV. of France, about 5000 were killed in ten years in single combat, and 14,000 others were similarly engaged. All France went mad upon the duel. Kings, popes, and bishops in vain fulminated against it. "At last," says Lord Herbert, the English ombassador, "there was scarcely a Frenchman deemed worth looking at who had not slain his man."

Infanticide has a strong tendency to become epidemic, of which we will mention one instance only. In one of the departments of France, about the close of last century, a girl killed her illegitimate child. The case created much excitement and interest, as there had not been a crime for very many years of that nature. Within twelve months, eleven others occurred in the same department, very similar in details.

No individual crime seems to have so strong a tendency to spread by example and imitation as *Suicide*.

"L'apparition épidémique du suicide," says M. Esquirol, "est un phénomène bien singulier. Dépend-elle d'une disposition cachée de l'atmosphère, de l'imitation qui le propage, de circonstances politiques qui bouleversent un pays, ou de quelque idée dominante favorable au suicide? Il est certain que cette apparition subite et passagère, mais en quelque sorte épidémique, appartient à des causes différentes."

Some of the illustrations which follow are extracted from Dr. Winslow's "Anatomy of Suicide," and also from M. Esquirol's essay on Suicide in the "Dict. des Sciences Médicales."

In the time of the Ptolemies, a stoic philosopher preached so earnestly and eloquently contempt of life and the blessings of death, that suicide became very frequent. The ladies of Miletus committed suicide in great numbers, because their husbands and lovers were detained by the wars! At one time there was an epidemic of drowning amongst the women of Lyons—they could assign no cause for this singular tendency—it was checked by the order that all who drowned themselves should be publicly exposed in the market-place. That at Miletus was stopped by a similar device—the ladies chiefly hung themselves—and the magistrate ordered that in every future case the body should be dragged through the town by the rope employed for the purpose, and naked. An ancient historian of Marseilles records that the girls of that city got at one time the habit of killing themselves when their lovers were inconstant!

The following passage is extracted from the "Anatomy of Suicide:"—

"Sydenham informs us, that at Mansfield, in a particular year, in the month of June, suicide prevailed to an alarming degree, from a cause wholly unaccountable. The same thing happened at Rouen in 1806; at Stuttgard, in 1811; and in the Valois, in the year 1813. One of the most remarkable epidemics of the kind, was that which prevailed at Versailles in the year 1793. The number of suicides within the year was 1300—a number out of all proportion to the population of the town."

Suicide not unfrequently accompanies epidemics of a bodily disease, such as pellagra. It is said that one-third of the victims of this affection commit suicide. Nostalgia is also a very frequent cause of this crime.

Closely connected with this subject is that of self-mutilation, a singular instance of which was related in the *Medical Times* some years ago; it is entitled

"An Epidemic of Voluntary Mutilations.—In the month of February, 1844, 350 men of the 3rd battalion of the 1st Regiment of the Foreign Legion were encamped at Sidi-bel Abbés, in the province of Oran. A soldier mutilated himself by a blow upon his wrist with the lock of his gun. Thirteen others inflicted a similar injury upon themselves within twenty days. None of these men would admit that the mutilations were voluntary; but all affirmed, that they arose from pure accident while cleaning their arms. It was not possible, in a single case, to discover a plausible motive to explain so strange a circumstance. The commanding officer, alarmed at this singular epidemic, and supposing it might extend, removed the camp some seven or eight leagues, to a place occupied by the 10th battalion of Chasseurs of Vincennes, commanded by M. Boëte. The astonishment of the officer commanding the Foreign Legion was great, when M. Boëte informed him that eight of his men had mutilated themselves in the same way, and nearly at the same time. The commanding officer and the surgeon both affirm, that there was no communication between the two camps. But even supposing that a communication had existed, it affords another example of the force of imitation."

We have deferred till the close of our list of the vices and crimes which disfigure humanity epidemically, that of Poisoning, partly because of its close connexion with the aspect of the present time, and partly because from its secret nature, the facilities which are afforded for its commission, and the difficulties in the way of its detection, it appears to us to exercise a more fearfully demoralizing influence upon society than any of those already noticed, dreadful as is the aspect of many of them.

"Early in the sixteenth century," says Mackay,* "this crime

* For many of the succeeding details we are much indebted to Mr. Charles Mackay's account of "The Slow Poisoners," in his "Memoirs of Extraordinary Popular Delusions," vol. ii.

seems to have gradually increased, till in the seventeenth it spread over Europe like a pestilence." An attentive consideration of the facts will show that this rapid spread quite naturally resulted from the well-known causes in operation—evil passions originating the crime, which then became popular, by temporary impunity, by impulse, by imitation, and by the publication of details, leading the public mind to dwell upon the subject, and gradually inducing a familiarity with the crime, and a proportionate contempt for human life. Many of these influences are even now rife, and the result is the harvest of crime which is constantly thickening around us. If the history of the past *be* the interpretation of the present, and the prophecy of the future, surely some useful lesson may be learnt by the accumulation of the experience of past ages.

Sporadic cases of poisoning occur very far back in history; but the first epidemic which we meet with is in Italy in the seventeenth century. Lebret, in his "Magazin zum gebrauche der Staaten Kirche Geschichte," relates that in 1659, Alexander VII. was informed by many of the clergy, that a number of young women had confessed to having poisoned their husbands, for various motives; no names were mentioned, but the authorities were directed to look out for these events. This caution resulted in the discovery of a society of young wives, who met nightly at the dwelling of an old woman called La Spara; and their business was to arrange the details of their poisonings. La Spara and four others were hanged; thirty were publicly whipped through the streets, and a great number were banished. Shortly afterwards nine others were hanged, "and many more, including young and beautiful girls" (Mackay), were whipped half naked through the streets of Rome. To these succeeded the notorious Tophania, the inventor of the "Aqua Toffana," now generally supposed to have been a solution of some neutral arsenical salt. This wretched creature carried on her horrible trade for above fifty years, selling poison to those who could afford to buy; but such was her sympathy, says Lebat, with those who were tired of their husbands, that she freely gave it to them, if they could not afford to pay. She was ultimately detected and strangled, after having confessed her crimes and her employers. The succeeding punishments for the time checked the mania.

About the same time, or a little after, a similar epidemic appeared in France. Between 1670 and 1680, Madame de Sévigné feared that Frenchman and poisoner would become synonymous, so frequent was the crime. The horrible series of murders perpetrated by Madame de Brinvilliers may be passed over as being well known; but it is especially interesting to trace their effects upon the public mind. We quote again from Mr. Mackay :—

"During the trial all Paris was in commotion. La Brinvilliers was the only subject of conversation. *All the details of her crimes were published, and greedily devoured; and the idea of secret poisoning was first put into the heads of hundreds who afterwards became guilty of it.* It was now (*i. e.*, after her execution and confession) that the mania for poisoning began to take hold on the popular mind. From this time to 1682, the prisons of France teemed with persons accused of this crime. We have already seen the extent to which it was carried in Italy—it was, if possible, surpassed in France—disgrace was, in fact, entailed, in the eyes of Europe, upon the name of Frenchman."

The criminals were detected ultimately, and many burned or hanged in 1679 ; but "for two years longer the crime continued to rage, and was not finally suppressed till the stake had blazed or the noose dangled for *upwards of a hundred individuals.*"

Hitherto we have had in England no such fearful epidemic as these, but are we not even now exposed to the droppings before the tempest? Do we not hear the growling of the thunder before the storm breaks in all its fury?

In the year 1845, a year memorable in our annals, the case of Tawell the Quaker, which is too well known to need recapitulation, excited much interest, and was the topic of almost exclusive comment for some time, even in those days of commercial madness. Poisoning was brought prominently before the public; and the mere accident by which detection was brought about, suggested to many minds the facility with which such crime could be accomplished, and *perhaps* escape detection. Whoever will take the trouble to examine the "Annual Registers" since that period, will find almost constant reference to the great increase of poisoning in Great Britain. Public indignation was greatly excited a few years ago at the revelations made concerning the burial-clubs: the number of the children that fell victims at this time is not to be ascertained, but was certainly great; and "we remember," says a vigorous writer in the *Express* of March 14th,—

"The sudden revelation of poisoning practices among the neglected poor in certain agricultural counties, where mothers had been taught, by the operation of the Corn Laws, to believe that the most loving office they could fulfil towards their children was to send them early from the pains of life to be 'better off with the Lord.' None of us are likely to forget that one very poor woman avowed, without any sense of guilt or shame, that she had thus dismissed to ease and plenty eight infants in succession by putting arsenic on her breasts.

We in 1856 seem threatened with the storm, of which these were but the preliminary drops; the crime of poisoning is brought prominently before us, it fills men's minds, and illustrations of it crowd our daily and weekly papers. But we must refrain—*these* cases are not yet matters of history—on the most important amongst them, the laws of our country, administered by those who rank amongst the wisest of her sons, have yet to pronounce their verdict, and we will not, even constructively, prejudge the matter.

The cumulative portion of our task is ended. To exhibit mind in its *contagious* aspect, we have passed in review not only those conditions of aberration which from their transitory nature may most strictly be considered as epidemics, but also those which having risen from small beginnings, have spread rapidly, and ultimately exercised a permanent influence upon the race. We have seen that in all its manifestations, Thought, Emotion, Expression, and Action, mind has a powerful action upon mind. The individual error or crime acts upon the mass by suggestion—the mass reacts upon the individual by intensifying every development of emotion. The tension of thought, which at first leads to any delusion, may be but slight; but when it takes hold upon numbers, each individual is affected by the combined force of these numbers. It is like the addition of plates to a galvanic battery, and the effect is almost like it, numerically proportionate. The man who timidly enunciates an opinion so long as it is but his own, will die in its defence when strengthened by the moral force of thousands. And this staunch adherence to any given view, is quite independent of whether it may be right or wrong, important or otherwise. Nothing can more strongly illustrate this position, than the persistency with which, when the witch-mania was fairly established, the victims of this delusion persisted in dying in support of their belief.

Our catalogue of error, folly, fanaticism, and crime, has been a long one; yet we have selected but a very small number from those with which all history abounds,—may we not say, of which almost all history consists? This would, however, be a profitless enumeration, if we could not deduce some general principle, as indicative of the causes of all the singular phenomena passed in review.

Granting the corrupt nature of man to be the primary source of all crime, we cannot fail to see that its development is favoured and fostered by the predominance of appetite and instinct over volition,—of imagination and impulse over reason and judgment. And what is this but the permanence of an *infantile* condition of mind? Children have appetites and instincts strong—reason undeveloped—passion unregulated. A proper system of education (strictly so called) has a tendency to substitute reason for instinct, to develop the former, to hold in check the latter. If this be neglected, or if it be misdirected, man will grow up a child in all but its innocence and its inability to do evil,—his appetites, impulses, and passions are strengthened by indulgence and lack of any restraining influence, his reason and judgment are null from disuse. In this state (and of how vast a majority of our fellow-creatures is this the condition) he is an easy prey to any class of ideas or emotions which may be presented to him —he receives them, adopts them, and imitates them, because he cannot analyze them—because they, perhaps, tend to the indulgence of the desire of the eye, or the lust of the flesh—because they flatter his pride—but most chiefly because uncultivated and uneducated man is *essentially mimetic.* Of the influence of morbid imitation in producing crime, many instances have already been given. Two very remarkable cases will be found in our Foreign Department, under the head of "Suicide amongst Children." Dr. Winslow, in his "Anatomy of Suicide," relates the following:—

"A criminal was executed not many years ago, in Paris, for murder. A few weeks after, another murder was perpetrated; and when the young man was asked to assign a reason for taking away the life of a fellow-creature, he replied, that he was not instigated by any feeling of malice, but, *after having witnessed the execution*, he felt a desire, over which he had no control, to commit a similar crime, and had no rest until he had gratified his feelings."

A similar instance occurred recently in one of our Northern counties, where the only reason which the murderer could give for cutting off the head of a child was, that W—— (mentioning the name of another notorious criminal) had done so before him. The following remarkable instance is also from Dr. Winslow's "Anatomy of Suicide:"—

"Some years ago, a man hung himself on the threshold of one of the doors of the corridor at the *Hôtel des Invalides*. No suicide had occurred in the establishment for two years previously; but in the succeeding fortnight, *five invalids hung themselves on the same cross-bar*, and the governor was obliged to shut up the passage."

"Last week, a boy, who had witnessed the execution of Baker, at Southampton, was found hanging by a rope from his mother's bed."—*Liverpool Albion*, Feb. 18, 1856.

It is needless further to multiply examples; the imitative instinct is perhaps the most powerful in our nature; and

"It is in homicidal mania that we look for the most striking illustrations of this mysterious form of cerebral disease. The instances on record of the dreadful exercise of this perverted instinct, under circumstances the most peculiar and afflicting, are numerous and well-authenticated, and the law is now well established among cerebral physiologists, that to persons thus diseased, the latent impulse—the lurking demon—is often forced into resistless action by the influence of a striking or notorious example. One startling and celebrated murder is the sure herald of several. The notoriety attracts to a congenial crime the diseased minds of thousands; a morbid sympathy is created; there is fascination in the gulph; the diseased propensity is stimulated, excited, and made to overwhelm both volition and reason. The last agency wanted is supplied to make the madness culminate."—*Medical Gazette.*

Love of notoriety is a strong incentive to crime.

"The man who was killed by attaching himself to a rocket, and he who threw himself into the crater of Mount Vesuvius, were no doubt stimulated by a desire for posthumous fame. Shortly after the suicide at the Monument, a boy made an unsuccessful attempt to poison himself; and on being questioned as to his motives, he said, 'I wished to be talked about, like the woman who killed herself at the Monument!'"

In the case of the man who cut the child's head off, mentioned above, a very striking feature was the desire for notoriety—the contemporary press has the following article:—

"While in the cell at the Town Hall, he was gratified when, by his mimicry or other means, he could attract the attention of persons in the office above. When being taken out on Monday, he anxiously inquired whether there were a good many people standing outside, intimating that he should shout out to them if there were; and on finding nobody standing about, he exhibited much disappointment. While in the cab, and also, after being placed in the railway carriage, he persisted in sitting close to the window, and seemed pleased at the slightest notice. His utter insensibility to the awfulness of the crime which he has committed, is, however, most strikingly illustrated by a piece of shocking levity in which he indulged also on Sunday. The attention of several of the police officers, who were in the receiving office, was attracted by bursts of merriment from the prisoners, and on looking in the cell-yard, the officers saw H— standing in a stiff, upright position, slowly turning his head backwards and forwards. In reply to an inquiry what it all meant, the prisoner said, 'I am only showing them how I shall look in waxwork, next fair.' This performance he went through a number of times during the day, complying unhesitatingly with every request to 'show them again.'"

Another powerful instinct is that of *impulse.* By this we mean an apparently irresistible tendency to the commission of a certain act, without motive, without any knowledge of the cause, but that the necessity to perpetrate it is most urgent.

A very striking instance of this is mentioned by Esquirol. A young girl, of unexceptionable morals and character, of mild and amiable deportment, acting as a nurse, one day met her mistress coming in from a walk, and requested to be dismissed the house. On being questioned as to her reasons, she said that every time she undressed the child, the temptation to kill it was almost irresistible, apparently stimulated by the sight of its white skin. This seems to ally this class of phenomena to those animal instincts and passions which are aroused by the sight of bright colours, as scarlet to the bull, &c.

The well-known case of Henriette Cornier, related by M. Marc, was of a similar nature, with this exception, that she accomplished her purpose, the impulse having proved too strong for her to overcome—the child was one to which she had always professed and felt extreme attachment.

All writers on the psychological relations of crime recognise, that in an otherwise sound mind this strong and occasionally irresistible tendency may suddenly occur, and depart again as soon as gratified, leaving the intelligence and the moral disposition in every respect unaffected. Instances of it occur very frequently after the public mind has dwelt for some time upon any given crime—yet it is altogether different in nature from the tendency to imitation, before noticed. Many of the subjects of it have sufficient warning given, to enable them to request to be restrained, or that the objects of their maniacal fury may be removed.

"Une jeune dame qui s'était retirée dans une maison de santé, éprouvait des désirs homicides dont elle ne pouvait indiquer les motifs. Elle ne déraisonnait sur aucun point, et chaque fois qu'elle sentait cette funeste propension se produire et s'exalter, elle versait des larmes, suppliait qu'on lui mit la camisole de force qu'elle gardait patiemment jusqu'à ce que l'accès, qui durait quelquefois plusieurs jours, fut passé."—Marc.

Without adducing further illustrations, we see plainly that a great proportion of mankind are, so far as their reason and intelligence are concerned, in the condition of *children*,—governed by instinct, appetite, and passion,—uncontrolled by conscience and judgment,—ready for any impression, prepared to tread any path marked out which leads to any indulgence, bodily or mental. The remedy for this is plain, palpable, and on the surface—difficult in detail, but ultimately practicable—a sound form of EDUCATION, secular and religious. *Education*, we say,—not *Instruction!*—nothing is more dangerous than knowledge to the mind without the capacity to make a proper use of it; then, indeed, it does but afford an additional facility for the commission of crime. It is through not carefully distinguishing between instruction and that sound education which should consist in the literal *educing* of the faculties of the mind, as a counteracting agency to the instincts, that Sir A. Alison has adopted his singular and almost paradoxical notions on the direct ratio between education and the increase of crime, as set forth in the following passage, and also in the introductory chapter to his recent history, at greater length:—

"Philanthropists anticipated, from this immense spread of elementary education, a vast diminution of crime, proceeding on the adage, so flattering to the pride of intellect, that ignorance is the parent of vice. Judging from the results which have taken place in Prussia, where instruction has been pushed to so great a length, this is very far indeed from being the case. On the contrary, though one of the most highly educated countries in Europe, it is at the same time one of the most criminal. On an average of three years, from 1st January, 1824, to 1st January, 1827, in Prussia, where the proportion of persons at school to the entire population was 1 in 7, the proportion of crime to the inhabitants was *twelve times* greater than in France, where it was 1 in 23. This startling fact coincides closely with what has been experienced in France itself, where the proportion of conviction to the inhabitants is one to 7285; and it has been found that, without one single exception in the whole eighty-four departments, the amount of crime is in the *inverse ratio* of the number of persons receiving instruction."—Alison's *History of Europe*, vol. v.

That a state-engine such as that of Prussia, little better than an instruction-mill, should produce results like these, is not surprising; but all the statistics of our own country, when *properly analysed*, show that crime and true education are perpetually in an inverse ratio; and we have the concurrent testimony of writers both upon psychology and crime, that it is chiefly defective or *perverted* education which is the source of mental aberration on the one hand, and of crime on the other. Mr. Hill, in his work on "Crime," places bad training and ignorance at the *head* of his causes of crime. He says—

"The great majority of those (criminals) that have come under my observation have been found to have been either greatly neglected in childhood, and to be grossly ignorant, or at least to possess merely a *quantity of parrot-like and undigested knowledge*, of little real value." —Hill *On Crime*, p. 36.

And again:—

"By direct education I need scarcely say that I do not mean the mere capability of reading and writing, but a systematic development of the different powers of the mind and body, the fostering of good feelings, the cultivation of good principles, and a regular training in good habits."—p. 48.

For much valuable information on this subject, we refer our readers to Mr. Hill's very excellent work, chapter 3rd.

An education which merely instructs will *encourage crime;* one which co-ordinates the faculties of the mind, which gives exercise to reason and judgment, at the same time that it represses *without ignoring* the instinctive part of man's nature, will elevate his position in the scale of creation, and turn those faculties to the service of his fellow-creatures, which otherwise would be employed to their destruction. If the emotions be constantly trampled down, and *invariably* subordinated to reason, they will in time assert their claims, and break forth in *insanity* or crime; if they be constantly indulged, the result will probably be the same. It is not by directing attention especially to them, but by elevating those tendencies of the mind which counterbalance them, that man will be brought nearer to the fulfilment of his high destiny, and his moral constitution be rendered less liable to those epidemics of folly and crime, upon which we have been commenting.

Deeply as these considerations affect the individual and societies, there are others which as closely involve the interests of the race; and these are so well and forcibly set forth by a recent writer in the *Express*, that we make no apology for quoting at length from his very philosophic article:—

"There is always something startling in a rapid succession of cases of the same kind of calamity or crime; and the witnesses of such a disclosure are apt to forget, in the strength of their emotions, that the experience of all ages should save us, on such occasions, from astonishment and dismay. Not only is there always a tendency in the criminal world, as in other worlds, to *modes* (to fashions based on sympathy and imitation), but there is a deeper cause for the existence of modes of suffering and of crime. . . . It is a fact, which has employed the pens of some thoughtful physicians and moralists, that changes in bodily functions and even structure attend on changes in civilisation, and that every important discovery in science is followed by new and strange human phenomena, individual and social. Very curious details may be found in medical literature on the subject of the varying physiological conditions which have attended the different periods of our civilisation. We have never met with a medical man who could or would say how it was that the women in Queen Elizabeth's time—the ladies of her court, for instance—could live as they did, and keep their health and attain old age. . . . The alimentary apparatus, with all that it involved, was then the strong and the weak point; and the nervous system is the strong and the weak point now. People could then digest like ostriches; but the abuse of the power led to 'surfeits,' fevers—inflammatory disorders of all kinds. People can now get a great deal more out of brain and nerve than brain and nerve were then trained to yield; but the complement of the case is, that we witness more nervous ailment and stranger phenomena of the nervous system than were ever distinctly observed before. Science has helped to alter the conditions of our life by a variety of new disclosures. Sir Charles Bell's great discovery in the matter of nervous structure has brought into light and prominence whole classes of diseases and liabilities; and the all-important reforms caused by science in the study and dissection of the brain have thus far thrown our practical methods of dealing with disease and certain orders of crime into confusion, rather than fitted us to treat them as wisely as the next generation may do. At the same time, there has been a vast development of the science of animal chemistry; and we are in the first astonishment at discovering how the curious mechanism of our bodies is sustained and kept going. Our condition is precisely that in which abnormal nervous states are most striking to us, and in which the subjects of food and poisons are interesting to the greatest number of people. If a wise student of history, secluded from the world, were told of the scientific and physiological conditions of the time, he would probably declare us to be liable to new and unaccountable manifestations through the nervous system, probably to a fashion of poisoning by new methods, and certainly to an epidemic credulity and suspicion about poisoning."

The writer then proceeds at considerable length to argue from these premises the necessity for taking these changes into consideration in deciding upon the phenomena of the present times, and urges most strongly caution in receiving prejudice as proof, and assertion as corroboration of crime.

Profoundly involved in the mysteries of our nature, and in those connected with the tidal progress of our race, these great *predisposing* causes of delusion and crime only admit of indirect influence by human agency.

There are others, of a more directly *exciting* character, which are dependent upon our social and political institutions, and which therefore admit of modification, if such can be pointed out, as likely to influence the spread of moral contagion in society. Our limits compel us to be very brief upon this most important topic. The evils to which we refer originate from the *Press*, the *Pulpit*, the *Bar*, the *Legislature*, and *Science*.

1. The great publicity given to the minutiæ of atrocious crimes in the public Press is undoubtedly a fruitful source of crime in this and other countries. The evil is a great and an admitted one: the remedy is yet to be discovered. There is always floating on the surface of society a numerous class of persons of questionable moral sense, ripe and ready for every kind of vice, eager to seize hold of any excuse for the commission of grave offences against the person and property. This class is generally more or less affected by the publication of the minute details of murder, suicide, and other crimes. To them such particulars are dangerously suggestive. They tend, as it were, to form the *type* of the moral epidemic, and to give form and character to the criminal propensities. Esquirol, and many others, complain bitterly of the effect of the public press in increasing the amount of cases of maniacal crime. We will not multiply instances, but select one only, as especially interesting in its evident origination from the publication of the details of another case. The following extracts are from the evidence given before the Coroner in the case of Mr. Dove of Leeds, accused of poisoning his wife with strychnine :—

"Mr. John Elletson, a pupil of Mr. Morley, proved that on several occasions he had had communications with Mr. Dove at the surgery. On one of these occasions, about a fortnight ago, he began by talking about Palmer's case. He said he believed strychnia could not be detected after death. I said I thought it could, and mentioned some of the tests. He asked me the effects of strychnia on man. The Coroner: Was anything said about antimony? Mr. Elletson: I think he saw the bottle, and said that was the poison that Palmer used.

"James Peacock said: I am surgery boy to Mr. Morley. I know the prisoner by coming for his wife's medicines. I have known him since December. I have been present in the surgery nearly every time he came. I was present about four or five weeks ago. Mr. Dove came for his wife's medicine. He looked at the bottles, and said, 'Tartrate of antimony,' and observed, 'I suppose this is what Palmer killed his wife with.' On the same shelf was strychnia, and he said, 'I suppose they can't test strychnia?' I said, 'Yes, they can.' He replied, 'They can test all mineral and vegetable poisons but strychnia.'

"Henry Harrison said: I am a dentist. I have known the prisoner for sixteen or seventeen months. I remember having had a conversation with him two months since about Palmer's case, at the New Cross Inn, South market. He sent for me about two months since. I read aloud Palmer's case in the bar in his presence. He said, 'Could I get him any strychnia?' I said, 'Not for the world.' I had another conversation with him about strychnia at the same house, about half-past two last Thursday. He sent for me to the same house, and I went there. He asked me, 'If they could detect a grain or a grain and a half of strychnia?' I said, 'Why, have you given your wife some?' He said, 'No, but I have spilt some.' (Sensation.)"

Can anything more strongly illustrate the evil tendency of the publication of scientific and other details? The particulars constantly retailed, also, in the papers, as to the state of health and mind, the deportment and general conduct of notorious criminals, are the strongest inducements to many weak-minded persons to take the same means of acquiring notoriety. Add to this, that not many days ago we met, in one of our most extensively circulated papers, with a popular account of the *precise method of making strychnine*, and we need say no more to show the fearfully evil influence which an unregulated press is calculated to have on society.

2. The influence which the Pulpit exerts is of two kinds, negative and positive—the lack of proper, and the actual existence of improper, teaching. On the former point, we shall allow the Church to speak for itself:—

"It is impossible to doubt, or to conceal, that very much of the preaching of the present day has been defective in those qualities which the character, temptations, and sins of the times require. There has been, in many quarters, plenty of vague generality, and semi-sentimentalism, but very little of definite practical teaching and intelligible counsel. What is called, *par excellence*, the preaching of 'vital godliness,' has dealt very little with the real life of men, women, and children, in detail, day by day, and hour by hour. Conventional language, conventional thought, and conventional feeling, have been excited and cultivated; but these are, in many instances, wholly ineffective, or inadequate for the real battle of life, with the world, the flesh, and the devil, in all their varied and ever-varying disguises, temptations, and deceptions. To what purpose is it to preach, Sunday after Sunday, on 'imputed righteousness,' to the man who is contemplating forgery to supply his extravagance; or upon 'justification by faith only,' to those who are about to ruin their friends or neighbours in order to sustain their own credit; or upon the 'errors of Popery,' to those who are knowingly selling adulterated articles, or using short weights and measures; or upon the doctrine of Predestination, to those who are ill-treating their wives, and bringing up their children like heathens? We fear that in many cases we have exchanged what was sneered at as mere 'moral preaching,' for something which, in its practical effects, allows a good deal of immorality to go on, unrebuked by the clergy or by conscience."—*English Churchman*, 28th Feb. 1856.

With regard to positively improper teaching, it must be acknowledged that there are few now, who, like that renowned street preacher mentioned by Mr. Villette, who exhorted his hearers to become like Jack Sheppard; but perhaps the following incident indicates a state of morbid craving after effect not less objectionable. For obvious reasons we mention no names, but vouch for the correctness of the occurrence. A wretched man, W——, committed in cold blood a most atrocious crime, for which he was afterwards executed. A minister visited him, and hoped that his counsels were not thrown away. On his return home he assembled his congregation and preached, in a style of by no means contemptible eloquence, a sermon upon the penitence and pardon of "this poor erring yet suffering fellow-creature"—depicted his tears and his sighs, and his reminiscences of his young days when he went to the Sunday School—the manner in which their joint petitions had ascended from that cold cell to the Throne of Grace; and all this, in a manner so acceptable to his audience, that very many were taken out in hysterics. It was not long before one of that district, if not that very congregation, was tried for a crime similar in nature, and for which he could give no reason, but that W—— had done so before.

3. With great caution would we comment upon the influence which the Bar may have upon the spread of crime. We are not prepared to suggest any remedy—our law recognises no man's guilt until it is proved, and all are equally entitled to such defence as the law allows. But knowing how powerful an incentive to crime is the love of notoriety, let any one glance over the impassioned address of Mr. Kelly to the court, in the defence of Frost, on a charge of high treason—the glowing eloquence of Mr. Phillips, labouring under the withering disadvantage of the confession of Courvoisier's guilt—the pathetic appeal of Mr. Robertson in favour of Alex. Alexander, tried for the crime of forgery—or the thrilling and soul-stirring peroration of Mr. Whiteside's defence of Smith O'Brien—and then let him consider whether to be thus spoken of, would not be, to hundreds, a strong incentive to go and do likewise.

4. The encouragement which the Legislature gives to crime is derived from the *uncertainty*, and in many cases the *insufficiency* of punishment—from the publicity and notoriety encouraged in such punishments (for it is a common saying, that one hanging produces twenty)—and from the growing unwillingness to inflict capital punishment even for the most atrocious crimes. On this subject, a writer already quoted, after commenting upon the duty of the authorities to repress and punish crime, has the following observations:—

"While we are upon this part of the subject, we cannot forbear referring to a very recent case in which, it appears to us, the Home Secretary has utterly set at nought such considerations as these, and the duty of increased faithfulness in punishing prevalent crimes. Contrary to every principle of law and justice, and to general expectation, the man who murdered his wife at a friend's house in the Minories, last Christmas, has been reprieved. Without impugning the Royal Prerogative in this matter, we boldly assert that Her Majesty has been very badly advised—that it was a most flagrant case, and that the lives of many wives will be thereby exposed to a greatly-increased danger. The man was a simple murderer—nothing more and nothing less—and as justly deserved death as nine murderers out of ten who have been executed since the Divine sentence of death for murder was first pronounced. A reprieve in such a case goes far to make murder a mere lottery, as to the infliction of death as its punishment. And this comparative impunity for wife-murder occurs at a time when not only murders of all kinds abound in the land, but when both secret and open brutality towards wives (and other women) has arrived at a pitch which, we believe, has no parallel in the previous history of this country. Mercy to man is murder to woman in such a case."—*English Churchman*, 28th Feb. 1856.

5. The uncertainty of Science, both mental and toxicological, is a fruitful source of evil. The public press teems with illustrations of this position perpetually; we have scientific evidence for the defence, and scientific evidence for the prosecution, almost as formally as we have counsel. The Staffordshire papers announce that Mr. Palmer's defence is to be *purely scientific!* On one of the most important points now before the public—the detection of a subtle and powerful poison—the most eminent men are at variance. That they should differ amongst themselves in the details of a science not yet perfected, is quite natural; but that these things should be allowed to go forth to the world, so that men may screen their enormous vices under the wing of science, is a phenomenon so monstrous as to be scarcely credible.* In the plea of insanity, also, the law is so vague, and the opinions of psychologists are so at variance, that whilst one man, who is only more accomplished in crime than his fellows, is acquitted as insane, we have occasionally the sad spectacle of a maniac dangling in a noose upon our gallows! These things are a disgrace to science, and *these* at least are susceptible of some alteration for the better. If there *be* three men in the kingdom upon whose opinion the nation and our rulers can depend, surely, if formed into a permanent commission to inquire into the state of mind of supposed lunatics, their verdict would be much more satisfactory than that of a jury puzzled by the conflicting and desultory statements of casual witnesses, medical or otherwise. If there *be* three men, who are capable of conducting an impartial chemical investigation, how much more weight and conviction would their unbiassed analysis carry to the minds of all men in disputed cases of poisoning, than are attained by the present defective and vicious system of professional evidence!

Our work is done. It is ever a painful task to dwell exclusively upon the delusions and crimes of mankind; but it is in the *aberrations* of intellectual and moral nature that (as in other sciences) we must seek the clue to their normal laws. We have attempted to trace these aberrations, and have here met constantly with the conviction that man, who has an *individual* responsibility, is the plaything not only of *his own* passions and instincts, but through the laws of his being, also of those of others. We have seen that through these same laws, and others of still more profound and complex operation, large masses are likewise subject to evil influence, from the caprices or vices of one. In attempting to trace the causes of these phenomena, we have ventured to intimate that our press has a liberty which amounts to

* A singular instance of scientific special pleading once came under our own notice. A case of poisoning by arsenic was under investigation; the poison was found in the stomach in a large quantity, but the chemist employed *for the defence* asked the writer of this paper, if he had ever heard of the fumes of arsenic, which had been used amongst the whitewash for the wall, acting as a poison, as he intended to found the defence upon the opinion that the deceased did not die from what had been taken into the stomach, but from that used upon the wall!!

license; that our spiritual teachers are lax in their duties; that science is prostituted to evil purposes; and that our legislature is not entirely free from the imputation of adding *its* quota to the encouragement of crime. All this forms a problem of vast importance to humanity. Wise and thoughtful men are looking earnestly into it, and attempting its investigation; and we, in this imperfect sketch, have but wished to add our mite to the endeavour, by inquiring into the history and conditions of the past, which is indeed "the interpretation of the present, and the prophecy of the future."

---

